# Influence of Firing Temperature on the Physical, Thermal and Microstructural Properties of Kankara Kaolin Clay: A Preliminary Investigation

**DOI:** 10.3390/ma13081872

**Published:** 2020-04-16

**Authors:** Muazu Abubakar, Ayyankalai Muthuraja, Dipen Kumar Rajak, Norhayati Ahmad, Catalin I. Pruncu, Luciano Lamberti, Ashwini Kumar

**Affiliations:** 1 Department of Mechanical Engineering, Bayero University, Kano 3311, Nigeria; muazumani2004@yahoo.com; 2 Department of Mechanical Engineering, Sandip University, Nashik 422213, MH, India; a.muthuraja2010@gmail.com; 3 Department of Mechanical Engineering, Sandip Institute of Technology and Research Centre, Nashik 422213, MH, India; dipen.pukar@gmail.com; 4 Faculty of Mechanical Engineering, Universiti Teknologi Malaysia, Skudai 81310, Johor Bahru, Malaysia; nhayati@mail.fkm.utm.my; 5 Department of Mechanical Engineering, Imperial College London, Exhibition Rd., London SW7 2AZ, UK; 6 Department of Mechanical Engineering, School of Engineering, University of Birmingham, Birmingham B15 2TT, UK; 7 Dipartimento di Meccanica, Management e Matematica, Politecnico di Bari, 70125 Bari, Italy; luciano.lamberti@poliba.it; 8 Department of Mechanical Engineering, National Institute of Tehnology, Jamshedpur 831014, JH, India; aknitjsr08@gmail.com

**Keywords:** Kancara kaolin clay, density, porosity, shrinkage, firing, mullite ceramics

## Abstract

In this study, natural deposits of Kankara kaolin clay were collected and investigated in order to determine physical, microstructural, thermal, and firing properties and assess clay’s suitability as starting material for various ceramic applications. Chemical analysis of the clay was performed using XRF. Mineralogical analysis and thermal analysis of the clay were conducted using XRD and thermogravimetric thermal analysis (TGA)/differential thermal analysis (DTA), respectively. In order to assess its ceramic behavior, the clay was fired at 900–1200 °C. Maturation characteristics of fired ceramics were assessed by measuring bulk density, apparent porosity, and shrinkage. It was found that main oxides in the clay are alumina, silica, and potassium oxide, while other oxides are present in trace quantities. Kaolinite, quartz, and illite are the phases found from the XRD results, while mullite ceramic phase formed at firing temperature above 1100 °C. Maturation tests showed that ceramic properties such as bulk density and shrinkage increase with temperature, while apparent porosity decreases with temperature. The results presented in this study prove that the clay is an appropriate material for producing traditional ceramics.

## 1. Introduction

Natural clay and clay minerals are important raw materials for ceramic industries. Clay and its minerals have been widely used as a raw material for building and construction industries, such as tiles and bricks [1]. The properties of clay that are of interest to ceramic industries are chemical composition, phases present, thermal properties, refractoriness, and strength after firing [2]. These properties are vital for the optimization of a clay deposit in ceramic industries. Consequently, critical attention should be paid to investigating the ceramic properties of clay deposits such as Kankara clay. Nigeria has largest deposits of clay in the African continent; they can be found almost in every state [3]. These clay deposits are mainly used for traditional production of small-scale ceramics such as pots and paints. A common characteristic of clay is its white color, a main feature of kaolin clay, which contains the mineral kaolinite with reddish brown color due to the presence of Fe_2_O_3_, a common impurity associated with kaolin clay deposits [4].

After firing, kaolin changes its properties to obtained mullite ceramics with improved hydrophobicity, density, low apparent porosity, good optical properties, and electrical properties [5]. During firing of kaolin, various processes take place, including evolution of physical combined water, which starts at a lower themperature than 100 °C. Heating further to a temperature range of 400–500 °C, removal of chemical combined water takes place, and the dehydroxylation of kaolinite mineral, resulting in the formation of an amorphous material called metakaolinite. Further heating to 900 °C results in the formation of primary mullite spinel, which subsequently crystallizes into mullite by heating to a temperature range of 1000–1100 °C [6]. From the available literature, Kankara clay is yet to be characterized in a comprehensive way with respect to chemical, mineralogical, thermal, and firing properties. Furthermore, there is limited information on the quality and potential use of Kankara clay as a ceramic material. In order to fill this gap, the present investigation focuses on assessing physical, mineralogical, thermal, and ceramic behavior of Kankara clay. The potentiality of Kankara clay for producing traditional ceramics also is investigated.

## 2. Materials and Methods

**Figure 1:**
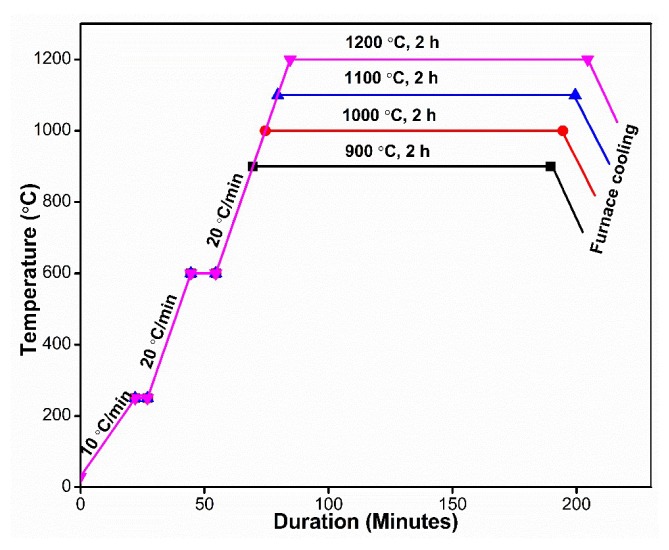
Sintering cycle of milled dried product of kaolin.

The clay analyzed in this study was chosen and sourced locally from Kankara (Local Government of Katsina State, Nigeria, Latitude 11°55′ N and Longitude 7°25′ E). Initially, the clay was washed with water in order to reduce the content of coloring impurities such as iron oxide. The material was then dried in an oven at a temperature of 100 °C for 3 days. After that, the dried clay was ground and sieved using a sieve shaker with sieves starting from 300 μm and progressively reduced to 50 μm with the aid of alumina balls [7]. The chemical composition of milled was verified using X-ray fluorescence (XRF). Milled clay was then compacted under uniaxial pressure of 60 MPa to A size 5 mm × 30 mm × 80 mm using an INSTRON machine (Instron 600DX, INSTRON, High Wycombe, UK) with 600 kN loading capacity. The glycerol acted as a binder during the compaction of the clay, and a drop of glycerol was added for this purpose. The compacted samples were then sintered at 900–1200 °C following the thermal cycle shown in Figure 1. After having been at the selected sintering temperatures for 2 h, samples were cooled in a furnace to room temperature. The phases in the raw clay and the sintered clay at 1200 °C were detected by means of XRD. The phases present in the developed materials were evaluated using an X-ray diffractometer (Siemens D5000; Siemens, Munich, Germany). XRD patterns were accessed with Cu K radiation (λ = 0.1504 Å) in 5° = 2θ = 50° in steps 0.05°.

Thermal behavior of each clay sample with 7.59408 mg mass was determined using differential and thermogravimetric thermal analysis (DTA/TGA) under nitrogen atmosphere from room temperature to 1100 °C at a heating rate of 10 °C/min. In this investigation, the firing and mechanical characteristics of the clay such as bulk density, apparent porosity, linear shrinkage, and flexural strength were obtained. The bulk density and the apparent porosity were determined according to Equations (1) and (2) [8,9]:

(1) $$ApparentPorosity=\frac{M_{c}-M_{a}}{M_{c}-M_{b}}\times 100$$

(2) $$BulkDensity=\frac{M_{a}}{M_{c}-M_{b}}\times \rho_{water}$$

where *M_a_* is the dry mass of the porous specimen; *M_c_* is the mass of the porous specimen soaked in water; *M_b_* is the mass of the porous specimen immersed in water; and *ρ* is the density of water at room temperature.

The shrinkage is calculated according to Equation (3) [10]:

(3) $$S_{t}=\frac{L_{p}-L_{f}}{L_{p}}\times 100$$

where *L_p_* is the length of the test specimen; *L_f_* is the fired length of test specimen; and *S_t_* is the total linear shrinkage after firing.

## 3. Results and Discussion

### 3.1. Chemical Composition

The chemical composition of the raw clay determined via XRF inspection (Table 1) shows that alumina and silica are the main oxides with highest percentage in the raw clay. Other oxides contained in the clay are potassium oxide (K_2_O), iron oxide (Fe_2_O_3_), calcium oxide (CaO), titanium oxide (TiO_2_), magnesium oxide (MgO), manganese oxide (MnO), and phosphorum oxide (P_2_O_5_). The loss of ignition (LoI) in the clay due to physical combined water, chemical combined water, and volatile matter accounts for 11.81%. Similar results for clays with high alumina and silica contents were reported in the literature [11,12,13].

### 3.2. X-ray Diffraction of Raw and Fired Clay

**Figure 2:**
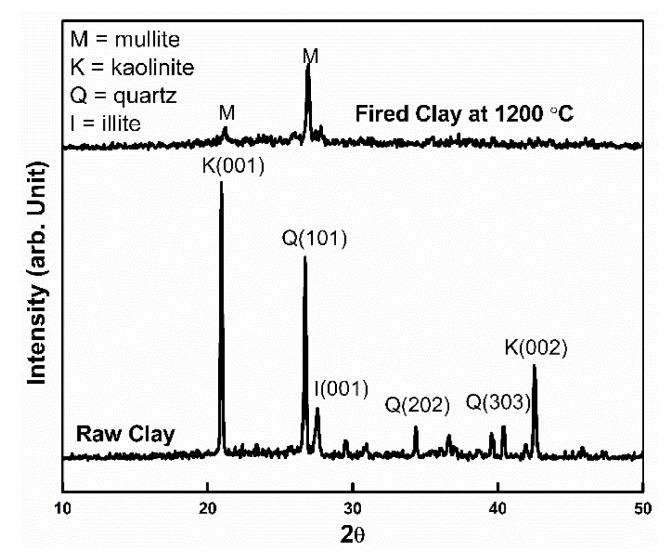
XRD patterns of raw clay and fired clay at 1200 °C.

The phase analysis of the clay was done using X-ray diffraction (XRD). The raw clay contains kaolinite as the major constituent phase, with some traces of illite and quartz (Figure 2). The relatively low intensity peak of the quartz in the kaolinitic clay indicates a significant presence of free silica [14]. The basal reflections of kaolinite and illite phases are characterized by (001) basal reflection plane, which corresponds to reflections found in clays (i.e., (001)), while for quartz basal reflection occurred in (101) plane. Similar observations were reported by [15,16,17].

The XRD pattern of fired clay at 1200 °C reveals the presence of mullite phase and disappearance of quartz and other phases. This result indicates that the kaoline phase transformed into mullite [18]. Since the presence of mullite phase increases thermal shock and creep resistance of fired clay [19], mullite materials have potential applications as ceramic substrates and refractories under high temperatures.

### 3.3. Thermogravimetric and Differential Thermal Analysis

**Figure 3:**
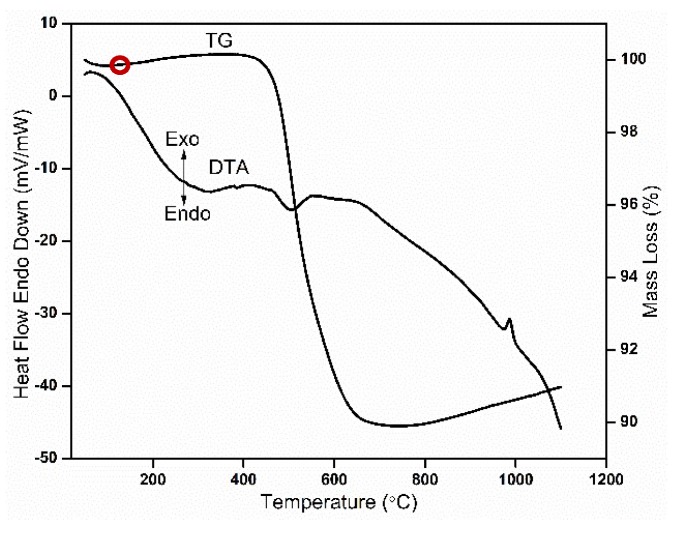
Thermogravimetric thermal analysis (TGA)/differential thermal analysis (DTA) thermal analysis of green bodies.

The thermal inspection of clay material by differential thermal analysis (DTA) and thermogravimetric (TG) analysis was carried out in inert atmosphere to 1100 °C at a heating rate of 5 °C/min and temperature (T) curves were simultaneously recorded. The DTA analysis of the clay shows various reaction peaks (endothermic and exothermic) during heating to 1100 °C. The first endothermic peak (highlighted by the red circle in Figure 3) occurs at a temperature between 70 and 100 °C; this peak is due to the removal of physical combined water present in the clay. The exothermic peak at 560 °C revealed that the complete transformation of kaolin to metakaolin was due to dehydroxylation (DHX) process. The exothermic peak at 980–1000 °C is due to conversions of metakaolin to spinel (Figure 3). The crystallization, densification and growth of mullite from spinel proceed at a higher temperature (above 1100 °C) [20].

The TGA analysis shows various reductions in mass caused by glycerol burn out and transformation of kaolin into metakaolin. Mass reduction for kaolin in the temperature ranges from 300 °C to 900 °C (about 10%). The less than 1% increase of mass loss observed between 100 and 400 °C and again between 900 and 1100 °C may be due to pressure fluctuations in purging of nitrogen at the beginning and the end of the analysis.

### 3.4. Bulk Density and Apparent Porosity

**Figure 4:**
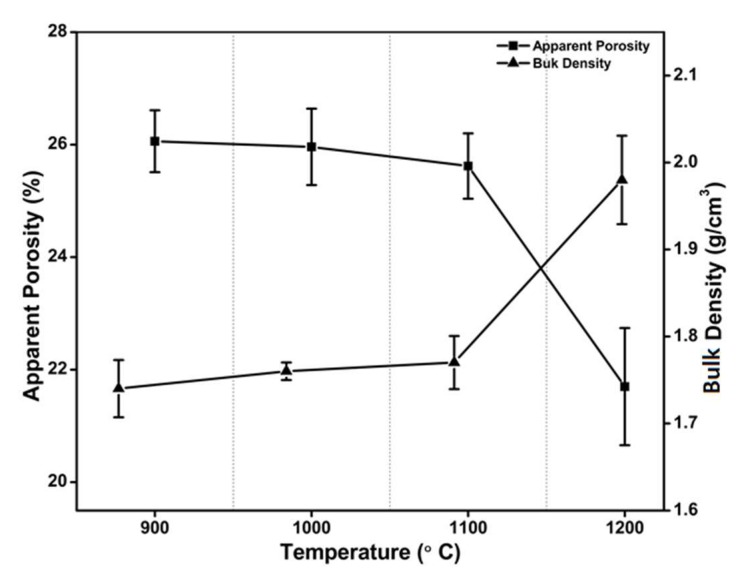
Variation of bulk density and apparent porosity with respect to firing temperature.

Variations of bulk density and apparent porosity of fired clay with respect to firing temperature are depicted in Figure 4. It can be seen that specimen bulk density changes marginally when firing temperature is in the range 900–1100 °C, while it increases sharply above 1100 °C. Figure 4 shows also that there is a very small reduction in apparent porosity as firing temperature increases from 900 to 1100 °C; porosity is markedly reduced for firing temperatures above 1100 °C.

Insensitivity of bulk density and apparent porosity to temperature for firing temperatures below 1100 °C, followed by the rapid variations observed above 1100 °C, can be explained with the presence of a glassy phase (Table 1) in the clay. This fact increases specimen densification as temperature goes beyond 1100 °C. A similar observation was reported by [2].

**Figure 5:**
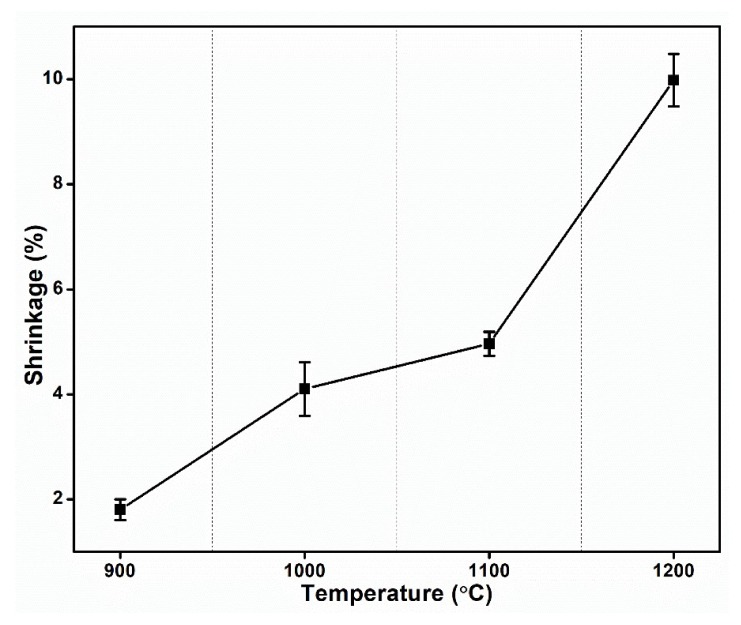
Variation of linear shrinkage with temperature.

Figure 5 shows that linear shrinkage has a similar behavior to apparent porosity and bulk density. This glassy phase forming substance includes quartz [21]. The linear shrinkage increases with firing temperature and reaches a peak at 1200 °C. This phenomenon can be explained with the formation of a liquid phase when heating temperature goes above 1000 °C.

**Figure 6:**
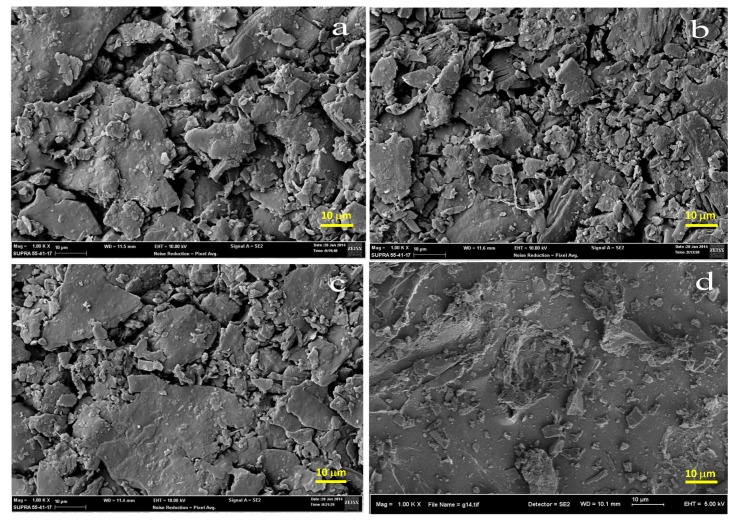
Fractured surfaces of fired clay for sintering temperatures of (**a**) 900 °C, (**b**) 1000 °C, (**c**) 1100 °C, and (**d**) 1200 °C.

Above this temperature, liquid surface tension and capillarity helps consolidate the particles, and hence there is a reduction in porosity. Similar observation was reported by [2]. Figure 6 depicts the morphology of specimen at firing temperatures of 900–1200 °C. From the micrographs, it can be seen that the consolidation of the fired clay increased with temperature.

Figure 6a shows poor consolidation of the fractured surface as aggregates of clay particles can be seen in the specimen heated at 900 °C. The consolidation becomes more evident as sintering temperature raises from 900 to 1000–1100 °C (Figure 6b,c). Figure 6d shows the fractured surface of the specimen sintered at 1200 °C: surface is more consolidated and presents well defined cracks. This is due to the formation of glassy phase [22]. Here, the presence of quartz was also confirmed by XRD analysis. The presence of clay particles in the fractured surface of the specimen sintered to 900 °C can be attributed to the formation of liquid glassy forming compound at temperatures below 1100 °C as reported by [20,23]. At higher temperatures, densification of the fired ceramic takes place via the formation of glassy phase.

## 4. Conclusions

In this experimental investigation, the phase composition, microstructure, and thermal properties of the Kankara kaolin clay were studied. The following conclusions can be drawn from the experiments.

- The XRD analysis of the sintered material shows the presence of the mullite phase and the disappearance of other phases. This result revealed the superiority in terms of thermal properties of the fired clay.
- (Physical properties): The sharp increase in bulk density occurring above 1100 °C sintering temperature and the continuous reduction in apparent porosity observed over the 900–1200 °C range can be explained in view of the consolidation of the clay.
- The linear shrinkage of the material increases by 500% as sintering temperature rises from 900 °C to 1200 °C. Therefore, the densification of the clay increased.
- (Thermal properties): The thermal analysis of the clay revealed the presence of an endothermic peak within 70–100 °C due to the removal of physical combined water present in the clay. An exothermic peak at 560 °C revealed that the complete transformation of kaolin to metakaolin is due to dehydroxylation (DHX) process.
- (Microstructural properties): SEM analysis of fractured surfaces showed poor consolidation of the fractured surface sintered at 900 °C. The consolidation becomes more marked as sintering temperature increases from 900 ° to 1200 °C and the fractured surface shows a more consolidated shape and the presence of well-defined cracks for the firing temperature of 1200 °C. This can be explained by the formation of the glassy phase.

## Figures and Tables

**Table 1:** Chemical composition of raw clay.

Compound	SiO_2_	Al_2_O_3_	K_2_O	Fe_2_O_3_	CaO	TiO_2_	MgO	MnO	P_2_O_5_
Concentration (%)	55.40	42.90	1.30	0.31	0.07	0.05	0.04	0.01	0.01
